# The burden of low back pain in BRICS: an analysis for the global burden of disease study 2021

**DOI:** 10.3389/fpubh.2025.1563260

**Published:** 2025-06-30

**Authors:** Li Shen, Wencong Cao, Yong Yu

**Affiliations:** ^1^School of Public Health, Hubei University of Medicine, Shiyan, China; ^2^Center of Health Administration and Development Studies, Hubei University of Medicine, Shiyan, China

**Keywords:** low back pain, burden of disease, BRICS, disability-adjusted life years, risk factors

## Abstract

**Background:**

To analyze the development of low back pain (LBP) disease burden in BRICS countries (Brazil, Russia, India, China and South Africa) from 1990 to 2021 and provide a scientific basis for China and its partner countries to carry out low back pain prevention and transnational medical cooperation.

**Methods:**

The burden of disease for low back pain was assessed by analyzing morbidity, prevalence, years lived with disability (YLD), and other disease burden indicators in BRICS countries in the Global Burden of Disease (GBD) Study from 1990 to 2021. The average annual percentage change (AAPC) was calculated using the Joinpoint regression model. Disease burden and risk factor attribution trends for patients with low back pain in BRICS countries were analyzed from 1990 to 2021. The Bayesian age-period-cohort (BAPC) model was used to predict and analyze future trends in low back pain globally and in BRICS countries.

**Results:**

The age-standardized incidence rate (ASIR) and age-standardized years lived with disability (ASYLD) for low back pain have generally decreased in China and India, while they have increased in Russia, Brazil, and South Africa. In 2021, the ASIR for low back pain in China was 2,342.46, and the ASYLD was 603.04, with decreases of 26.20 and 26.03%, compared to 1990. In Russia, the ASIR for low back pain in 2021 was 4,529.36, and the ASYLD was 1,206.23, an increase of 16.75 and 15.62% over 1990. In Brazil, the ASIR in 2021 was 3,873.61, and the ASYLD was 1,034.20, representing increases of 19.97 and 21.84%, respectively, compared to 1990. In 2021, the ASIR and ASYLD for low back pain are rising in South Africa, at 2,753.32 and 693.46, with the indices of the increasing trends being 5.46 and 3.04%, respectively, compared to 1990. All the differences observed were statistically significant (*p* < 0.001). India’s ASIR for 2021 is 2,816.31, down 5.70% from 1990, with no statistically significant trend change (*p* = 0.634), and the ASYLD is 714.00, a decrease of 8.83% compared to 1990, with no statistically significant trend change (*p* = 0.322). In BRICS countries, males with low back pain have lower rates of ASIR and ASYLD than females, and the ASIR and ASYLD in BRICS populations increase with age, peaking in the 70+ age group.

**Conclusion:**

From 1990 to 2021, the burden of low back pain declined in China and India, but morbidity and disability-adjusted life years (DALY) remained high. Russia, Brazil, and South Africa are on the rise, and health research on LBP needs to be intensified. Females and the older adult are at high risk of low back pain. Occupational ergonomics and high body mass index (BMI) are major risk factors affecting BRICS countries. The global incidence of LBP from 1990 to 2030 presents a significant downward trend in the future for males and a slight upward trend for females, with an overall global decline.

## 1 Introduction

Low back pain is one of the most common diseases affecting people’s health. It has complex etiology and many influencing factors, and has become the main cause of work absenteeism and activity limitation in the world today (1–3). According to China’s Burden of Disease Study from 2005 to 2017, low back pain was one of the most common diseases in China in terms of YLD loss due to non-fatal diseases in 2017 (4, 5). Indicators of low back pain in Russia have remained high for years, and medical delays and occupational conditions are important factors contributing to the burden of LBP (6, 7). ASIR is the age-standardized incidence rate which can indicate the patient’s illness condition and ASYLD is the age-standardized years lived with disability which can calculate the indicators of the loss of healthy life years caused by disability due to various diseases. India’s indicators are similar to those of China, with higher disease burden indicators early on. However, significant progress has been made in improving the overall burden of disease, with ASIR and ASYLD being the lowest among the five countries (8–10). In South Africa, the financial burden of low back pain remains high, with outpatient costs accounting for the largest proportion of total expenses and analgesics being the primary intervention strategy, constituting more than half of all outpatient expenses (11, 12). Brazil is the only country among the five with a growing disease burden, as well as a high incidence and heavy burden of low back pain. From 1990 to 2017, the prevalence increased by 26.83%, and the rise in burden is mainly related to population growth dynamics and the intensification of aging, while the LBP is one of the top three causes of ASYLD (13–15). The comprehensive MR study provides evidence that Weight control should be considered in populations with obesity to reduce the risk of LBP, sleep disturbance is common in patients with LBP, the intensity of back pain was only weakly associated with sleep disturbance, suggesting that other factors contribute to sleep problems for LBP patients, heavy workload and the accumulation of loads or frequency of lifts were moderate to strong risk factors for LBP, the occurrence of LBP is related to the nature and intensity of the physical activities undertaken (16–18).

Meanwhile, the globalization process has prompted countries to address global health governance as a major strategic concern, with the BRICS countries, represented by Brazil, Russia, India, China, and South Africa, playing an increasingly important role in global health governance (19, 20). This research analyzes the current situation and trends in the burden of low back pain in China and other BRICS countries from 1990 to 2021, intending to inform policy decisions on disease prevention and control in China and its partner countries.

## 2 Materials and methods

### 2.1 Data source

Lower back pain is very common. It often results from a strain (injury) to muscles or tendons in your back. Other causes include arthritis, structural problems and disk injuries. The data from the Global Health Data Exchange database,^1^ extracts incidence, prevalence, YLD distribution, and corresponding standardized indicators of low back pain in global and BRICS countries for 1990–2021. Low back pain is classified and coded according to the International Statistical Classification of Diseases and Related Health Problems, Version 10, code M54.503.

### 2.2 Statistical methods

Incidence and DALY for GBD were processed using R (4.2.1) software and analyzed for standardized data such as ASIR, ASYLD, and population attributable fraction (PAF). Joinpoint (5.0.2) regression modeling software was used for the analysis. The software is designed to analyze trend data and adjust the model based on the number of connections allowed in the data, testing whether the connections between the minimum and maximum values are statistically significant. Trends in the burden of disease for low back pain in the BRICS countries from 1990 to 2021 were analyzed using their regression models for average annual percent change and their 95% confidence intervals (95% CI) (21). The test level is *α* = 0.05. Attribution analysis of risk factors and joinpoint regression analysis was conducted using R (4.2.1) software, along with predictive analysis through the Bayesian age-period-cohort (BAPC) model.

## 3 Results

### 3.1 The overall burden of low back pain in BRICS countries in 1990 and 2021

In 1990, standardized morbidity and YLD rates ranked equally among the BRICS countries, with Russia having the highest indices for ASIR and ASYLD, followed by Brazil, China, India, and South Africa. In 2021, these indicators remained unchanged, with Russia maintaining the top positions in ASIR and ASYLD, followed by Brazil, South Africa, China, and India. In 2021, China, Russia, Brazil, India, and South Africa were ranked 200th, 17th, 54th, 146th, and 157th in the ASIR global rankings, and 184th, 21st, 51st, 151st, and 166th in the ASYLD rankings. Refer to Tables 1, 2.

**Table 1:** Ranking of BRICS countries in terms of overall ASIR severity, 1990–2021.

ASIR	1990	Global ranking	2000	Global ranking	2010	Global ranking	2021	Global ranking
China	3174.26	69	2446.73	200	2341.88	202	2342.46	200
Russian Federation	3879.4	22	3763.39	37	3780.2	34	4529.36	17
Brazil	3228.92	60	3254.03	60	3225.77	61	3873.61	54
India	2986.68	101	2457.69	198	2421.32	199	2816.31	146
South Africa	2610.70	187	2490.57	194	2463.92	195	2423.99	157

**Table 2:** Ranking of BRICS countries in terms of overall ASYLD severity, 1990–2021.

ASYLD	1990	Global ranking	2000	Global ranking	2010	Global ranking	2021	Global ranking
China	815.2	111	618.92	203	592.19	204	603.04	184
Russian Federation	1043.27	42	1007.04	46	1014.69	43	1206.23	21
Brazil	848.81	91	858.82	86	853.28	85	1034.20	51
India	783.18	128	625.52	202	618.04	201	714.00	151
South Africa	672.99	193	636.13	196	628.22	199	693.46	166

### 3.2 Overall development results of low back pain ASIR and ASYLD in BRICS countries from 1990 to 2021

**Figure 1:**
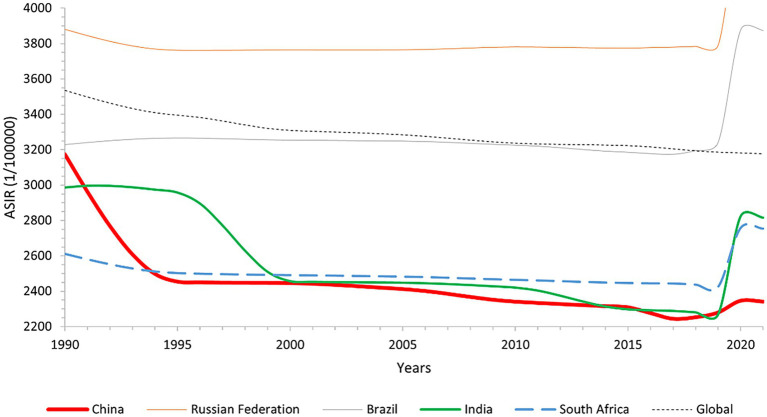
Line graph of global and BRICS overall ASIR changes, 1990–2021.

**Figure 2:**
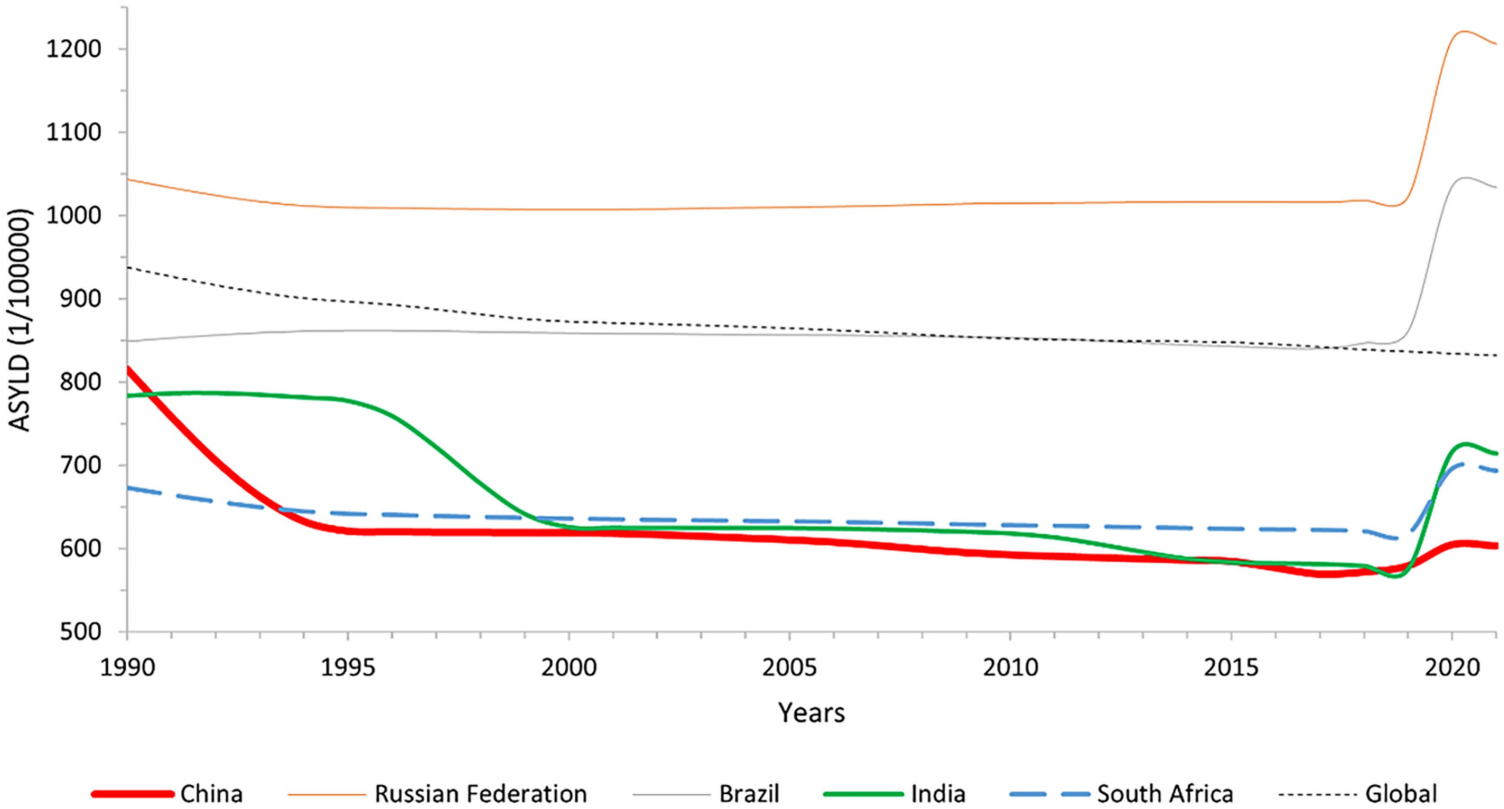
Line graph of global and BRICS overall ASYLD change, 1990–2021.

In 2021, China’s ASIR for low back pain has a decrease of 26.20% from 1990, and the ASYLD has a 26.03% decrease from 1990. The ASIR in Russia has an increase of 16.75% compared to 1990, and the ASYLD has a 15.62% rise from 1990. In Brazil, the ASIR in 2021 has an increase of 19.97% from 1990, while the ASYLD has an increase accounting for 21.84% over 1990. The ASIR for low back pain in South Africa in 2021 has an increase of 5.46% compared to 1990, and its ASYLD has a 3.04% increase from 1990. All the differences were statistically significant (*p* < 0.001). India’s ASIR has a decrease of 5.70% from 1990 (*p* = 0.634), and its ASYLD has a decrease of 8.83% compared to 1990 (*p* = 0.322); however, neither trend is statistically significant. As depicted in Figures 1, 2, and Table 3.

**Table 3:** AAPC of ASIR, ASYLD for different gender groups in BRICS overall, 1990–2021.

Country	ASIR (1/105)			ASYLD (1/105)		
	Male	Female	Subtotal	Male	Female	Subtotal
China						
1990	2738.75	3621.49	3174.26	697.09	936.36	815.20
2021	1901.62	2779.16	2342.46	488.36	716.15	603.04
AAPC	−1.139	−0.792	−0.938	−1.106	−0.799	−0.924
95% CI	−1.284, −0.993	−0.925, −0.659	−1.031, −0.844	−1.265, −0.947	−0.949, −0.648	−1.033, −0.815
*t*-value	−15.243	−11.641	−19.639	−13.599	−10.357	−16.527
*p*-value	<0.01	<0.01	<0.01	<0.01	<0.01	<0.01
Russian Federation						
1990	3233.49	4394.20	3879.40	829.91	1207.17	1043.27
2021	3620.62	5287.62	4529.36	922.7	1433.06	1206.23
AAPC	0.574	0.724	0.623	0.443	0.696	0.607
95% CI	0.389, 0.76	0.498, 0.95	0.403, 0.844	0.377, 0.509	0.474, 0.917	0.385, 0.828
*t*-value	6.086	6.304	5.558	13.133	6.175	5.385
*P*-value	<0.01	<0.01	<0.01	<0.01	<0.01	<0.01
Brazil						
1990	3038.91	3409.01	3228.92	794.89	899.70	848.81
2021	3036.49	4645.45	3873.61	793.56	1253.85	1034.2
AAPC	0	1.037	0.599	0.002	1.115	0.652
95% CI	−0.02, 0.02	0.7, 1.376	0.388, 0.81	−0.023, 0.026	0.758, 1.473	0.431, 0.873
*t*-value	0.005	6.041	5.577	0.133	6.150	5.799
*P*-value	0.996	<0.01	<0.01	0.894	<0.01	<0.01
India						
1990	2204.85	3840.95	2986.68	554.60	1032.61	783.18
2021	1800.12	3838.04	2816.31	444.66	983.77	714.00
AAPC	−0.616	0.07	−0.079	−0.676	−0.016	−0.195
95% CI	−0.862, −0.369	−0.593, 0.738	−0.406, 0.248	−0.938, −0.413	−0.475, 0.446	−0.579, 0.191
*t*-value	−4.883	0.207	−0.476	−5.030	−0.068	−0.990
*P*-value	<0.01	0.836	0.634	<0.01	0.946	0.322
South Africa						
1990	2591.57	2610.80	2610.70	663.89	675.77	672.99
2021	2228.95	3214.92	2753.32	557.21	811.62	693.46
AAPC	−0.525	0.81	0.343	−0.61	0.744	0.173
95% CI	−0.627, −0.423	0.493, 1.127	0.214, 0.473	−0.733, −0.487	0.426, 1.063	0.159, 0.187
*t*-value	−10.059	5.025	5.209	−9.694	4.598	24.345
*P*-value	<0.01	<0.01	<0.01	<0.01	<0.01	<0.01

### 3.3 Disease burden of low back pain among gender groups in BRICS countries from 1990 to 2021

From 1990 to 2021, the largest declines in ASIR and ASYLD for males occurred in China, decreasing by 30.57 and 35.74%, while the highest increases in female ASIR and ASYLD were in Brazil, rising by 36.27 and 39.36%, respectively. Refer to Table 3.

### 3.4 Burden of low back pain disease in the BRICS population of different ages from 1990 to 2021

ASIR and ASYLD for low back pain in the BRICS population increased with age group, with peak levels observed in the 70 + age group. The country with the highest ASIR in all age groups in 2021 was Russia. Russia also has the highest indicator of ASYLD, with the AAPC arranging the groups in increasing order of age: 0.785 (95% CI 0.563, 1.007), 0.852 (95% CI 0.562, 1.143), and 0.763 (95% CI 0.58, 0.946). From 1990 to 2021, Russia was the country with significant shifts in the trends of ASIR and ASYLD in the 15–49 age group, with a rate of 23.77 and 21.15%. In the 50–69 age group, ASIR and ASYLD showed the greatest trend of change, as did Russia, with 27.09 and 36.54%, respectively. Brazil leads in ASIR and ASYLD trends in the 70+ age group, according to rates of 37.82 and 42.15%. It is presented in Table 4.

**Table 4:** AAPC for ASIR, ASYLD for different age groups in BRICS overall, 1990–2021.

Country	ASIR (1/105)			ASYLD (1/105)		
	15–49	50–69	70+	15–49	50–69	70+
China						
1990	2653.31	6494.05	9358.78	699.49	1706.20	2399.79
2021	2294.29	5017.36	6782.61	596.44	1352.31	1706.32
AAPC	−0.408	−0.736	−1.01	−0.455	−0.638	−1.059
95% CI	−0.595, −0.221	−0.89, −0.582	−1.112, −0.907	−0.614, −0.296	−0.756, −0.52	−1.253, −0.864
*t*-value	−4.268	−9.320	−19.133	−5.597	−10.569	−10.610
*P*-value	<0.01	<0.01	<0.01	<0.01	<0.01	<0.01
Russian Federation						
1990	3676.55	7534.31	10753.82	995.81	2069.14	3041.13
2021	4550.62	9575.02	13037.79	1206.47	2618.32	3699.91
AAPC	0.853	0.833	0.766	0.785	0.852	0.763
95% CI	0.604, 1.102	0.542, 1.124	0.673, 0.859	0.563, 1.007	0.562, 1.143	0.58, 0.946
*t*-value	6.742	5.638	16.214	6.954	5.772	8.181
*P*-value	<0.01	<0.01	<0.01	<0.01	<0.01	<0.01
Brazil						
1990	3297.76	5815.47	6554.24	888.27	1580.29	1718.96
2021	4034.00	7331.14	9032.82	1097.17	2040.07	2443.48
AAPC	0.655	0.81	1.142	0.663	0.889	1.235
95% CI	0.477, 0.832	0.536, 1.084	0.779, 1.506	0.491, 0.835	0.599, 1.181	0.857, 1.614
*t*-value	7.260	5.810	6.186	7.588	6.017	6.434
*P*-value	<0.01	<0.01	<0.01	<0.01	<0.01	<0.01
India						
1990	2728.83	6068.75	7684.90	725.85	1637.98	1992.41
2021	2505.20	6117.66	7570.99	633.75	1643.31	1867.31
AAPC	−0.237	0.09	−0.046	−0.323	0.082	−0.143
95% CI	−1.202, 0.737	−0.112, 0.293	−0.258, 0.167	−0.877, 0.235	−0.175, 0.34	−0.352, 0.066
*t*-value	−0.479	0.874	−0.424	−1.136	0.623	−1.345
*P*-value	0.632	0.382	0.672	0.256	0.533	0.179
South Africa						
1990	2079.44	5790.81	7497.42	534.90	1522.05	2000.83
2021	2333.14	6321.18	8312.7	585.21	1647.5	2113.67
AAPC	0.472	0.485	0.572	0.37	0.393	0.288
95% CI	0.329, 0.616	0.348–0.623	0.392, 0.752	0.3, 0.44	0.16, 0.627	0.213, 0.364
*t*-value	6.472	6.944	6.253	10.377	3.310	7.488
*P*-value	<0.01	<0.01	<0.01	<0.01	<0.01	<0.01

### 3.5 Attributable disease burden of risk factors for low back pain in BRICS countries from 1990 to 2021

From 1990 to 2021, the number of patients with low back pain related to occupational ergonomics in China declined, while the number of patients with other risk factors increased in the BRICS countries. A high BMI was the leading factor in the rapidest growth. Occupational ergonomics remains the biggest risk factor in China, Brazil, and India. In Russia and South Africa, the biggest risk factor was a high BMI. Refer to Table 5.

**Table 5:** Attributable burden of disease for risk factors for low back pain in the BRICS countries, 1990–2021.

Variable	ASYLDs/104 Year	PAF/%
China		
Occupational ergonomic factors		
1990 year	348.67	40.7%
2021 year	331.41	29.3%
Change rate/%	−4.95	−28.01
Smoking		
1990 year	128.45	15%
2021 year	183.70	16.3%
Change rate/%	43.01	8.67
High BMI		
1990 year	15.55	1.8%
2021 year	106.20	9.4%
Change rate/%	582.96	422.22
Russian Federation		
Occupational ergonomic factors		
1990 year	31.23	17.8%
2021 year	32.67	13.9%
Change rate/%	4.61	−21.91
Smoking		
1990 year	32.64	18.6%
2021 year	39.55	16.8%
Change rate/%	21.17	−9.68
High BMI		
1990 year	13.37	7.6%
2021 year	43.48	18.5%
Change rate/%	225.21	143.42
Brazil		
Occupational ergonomic factors		
1990 year	24.90	23.3%
2021 year	53.20	20.9%
Change rate/%	113.65	−10.30
Smoking		
1990 year	22.26	20.9%
2021 year	30.73	12.1%
Change rate/%	38.05	−42.11
High BMI		
1990 year	4.96	4.6%
2021 year	39.72	15.6%
Change rate/%	700.81	239.13
India		
Occupational ergonomic factors		
1990 year	154.13	30.5%
2021 year	205.94	21%
Change rate/%	33.61	−31.15
Smoking		
1990 year	43.80	8.7%
2021 year	64.00	6.6%
Change rate/%	46.12	−24.14
High BMI		
1990 year	7.32	1.4%
2021 year	55.45	5.7%
Change rate/%	657.51	307.14
South Africa		
Occupational ergonomic factors		
1990 year	2.98	16.1%
2021 year	4.46	11.9%
Change rate/%	49.66	−26.09
Smoking		
1990 year	3.77	20.5%
2021 year	3.86	10.3%
Change rate/%	2.39	−49.76
High BMI		
1990 year	1.34	7.3%
2021 year	7.35	19.6%
Change rate/%	448.51	168.49

### 3.6 Global research on the prediction of low back pain, 1990–2030

**Figure 3:**
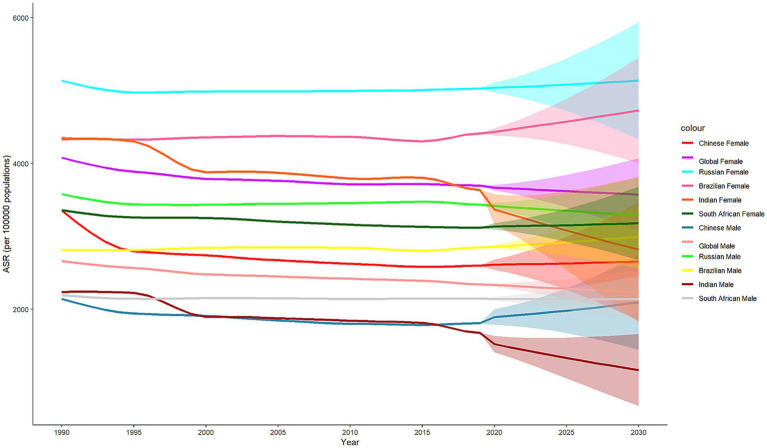
Axis plot of standardized incidence rates for the global and BRICS all-sex BAPC prediction model for low back pain, 1990–2030.

The global incidence of LBP from 1990 to 2030 presents a significant downward trend in the future for males and a slight upward trend for females, with an overall global decline. In the future, there will be a moderate increase in the number of males, along with some improvement. However, the general trend is increasing, particularly among females. The burden of low back pain will be greater and more severe in females than in males. In China, Brazil, and South Africa, the overall and gender prevalence of low back pain is expected to increase in the future. In Russia, the incidence of low back pain is decreasing in males and increasing in females. In India, the prevalence of both general and gender-specific diseases is declining. As depicted in Figure 3.

## 4 Discussion

From 1990 to 2021, the ASIR and ASYLD of low back pain in China decreased significantly, representing the largest reduction compared to the other five countries, leading to a substantial improvement in the overall burden of low back pain. Research into new and effective treatments, as well as the strengthening of various occupations—such as optimizing work hours, improving work environments, and raising awareness of different risk factors—is a key contribution to significantly reducing China’s burden of LBP (22–24). These efforts should continue to be prioritized and further advanced in the future. The prevalence of low back pain in Russia needs to be reassessed, as it hampers the development and implementation of effective measures. Crucial factors contributing to the rising burden of LBP in Russia include the lack of timely medical treatment and challenges in the occupational environment (6, 7). Additionally, during targeted drug research, greater emphasis should be placed on disseminating information about LBP and raising national health awareness. Improving the burden of disease in India depends on tailored and professional policies, targeted prevention and treatment strategies for high-risk groups, such as women, farmers, and grassroots workers. It also emphasizes the importance of promoting universal yoga and other national sports, as well as advancing research into tools for pain assessment, and data collection by category (8–10). In the future, India is expected to make significant progress in researching related diseases and play a crucial role in sharing experiences related to prevention and treatment. South Africa is responding positively to global initiatives to address low back pain as a major public health challenge in the country. While some progress has been made in LBP research, the financial burden remains high and there are still some issues to be resolved (11, 12). Efforts should be made to take appropriate measures, focusing on disease prevention and control policies, while ensuring international cooperation to minimize economic costs. In Brazil, ASIR growth is modest, while ASYLD is increasing. The country is experiencing significant changes in the epidemic of diseases, leading to an increasing number of people requiring long-term care (13–15). Therefore, it is crucial to focus on the burden of non-fatal health conditions, with particular emphasis on the prevention and treatment of low back pain, which should be prioritized as an important area of clinical research. Overall, Russia and Brazil have a higher burden of LBP than the other three countries, highlighting the need to strengthen the exchange of experiences with these nations.

The prevalence of low back pain in BRICS countries varies significantly by gender and age groups. In these five countries, the ASIR and ASYLD indicators are generally lower for males than for females. This discrepancy is closely related to female occupational factors and the time spent in manual labor and is universal (25, 26). For example, occupations with a high percentage of females include nursing and similar fields, which recommend strengthening exercises, reasonable rest, and reducing chronic strain injuries. In 2021, among the five countries, Russian females had the highest ASIR and ASYLD, while males had the highest ASIR (6). In order to mitigate the impact of occupational factors and raise awareness, workplace reforms and better practices are necessary. Brazil has the highest rate of male ASYLD and should focus on population growth and aging (13). Both of the aforementioned countries need to further strengthen relevant research to alleviate the burden of low back pain. The ASIR and ASYLD in BRICS populations increase with age, with peaks in the over-70s age group. The disease burden of low back pain is particularly severe in older adults, as it is a common and disabling condition. This often restricts physical function, impairs quality of life, and can lead to mobility problems, imbalances, and an increased risk of falls (27, 28). Russia has the highest prevalence and DALY across all age groups among the five countries, and research into targeted therapy is currently an effective way to reduce the burden of low back pain (29). In India, there has been significant progress in managing LBP among individuals aged 15 to 49. It is mainly due to the popularity of exercises such as yoga among young people [15]. In addition, China has significantly reduced the relative burden of low back pain for people aged 50–69 and those over 70. This improvement reflects not only advancements in healthcare and health promotion but also rapid developments in research on the factors affecting the older adult population (30). Among non-BRICS countries, the United States has adopted the method of increasing medical insurance for low back pain to reduce its burden, and the United Kingdom has emphasized effectiveness of physical treatments for back pain in primary care (31, 32). There are differences in the prevalence of LBP between young people and the older adult, and the older adult tend to have a higher prevalence. There are differences in the prevalence of LBP among workers in BRICS countries and non-BRICS countries. The differences are often determined by the economic level of the countries. Countries with a high economic level tend to have a lower incidence of LBP (4).

Controllable risk factors, such as occupational ergonomics, smoking, and a high BMI, are the main causes of the burden of low back pain. The burden of attributing risk factors to LBP varies in degree and trend, while the burden of disease caused by occupational ergonomics is decreasing. Smoking and a high BMI are major risk factors for low back pain, and the burden of the disease is increasing. This may be related to higher smoking rates, poor eating habits, inadequate workplace design, and highly stressful work environments. It is suggested that China enhance its awareness of the possible rebound in the incidence of low back pain in the future, further implement attention to high-risk occupational groups and continue to develop advanced treatment methods for low back pain. For instance, low-intensity exercise courses such as Tai Chi and Baduanjin can be incorporated into community older adult activities, and home renovation subsidies (such as anti-slip bathrooms) can be provided for home-based older adult care. At the same time, the prevention of low back pain can be included in the “Occupational Health Protection Action,” and the problem of prolonged sitting in the working system can be strictly investigated. Moreover, appropriate traditional Chinese medicine techniques such as acupuncture and massage can be promoted in public hospitals and covered by medical insurance (33). It is suggested that Russia reduce its excessive reliance on painkillers, make reasonable rest and work arrangements for people with high-intensity labor, and strengthen medical cooperation with other BRICS countries. At the same time, efforts should be made to enhance protection in cold regions. For instance, winter waist warmth measures (such as belt guards) should be promoted to reduce muscle stiffness caused by cold. Rehabilitation centers should be added in the Arctic region to provide physical therapy services. The intervention for low back pain should be combined with alcohol dependence treatment to prevent alcohol abuse from aggravating pain (34). It is suggested that Brazil pay more attention to the older adult group suffering from low back pain, and propose strategies to address the increasingly serious problem of low back pain while solving the aging issue. Training community doctors to promote primary management of low back pain (such as non-pharmacological treatments), reducing excessive reliance on specialized medical care, incorporating low back pain screening and rehabilitation guidance into family health plans, promoting inter-work stretching exercises and the use of ergonomic tools for manual workers in agriculture, construction and other industries, and requiring enterprises to provide ergonomic chairs and working environments. Finally, knowledge about the prevention of low back pain (such as the correct posture for carrying heavy objects) can also be promoted through social media (35). It is suggested that India accelerate the replacement of backward medical methods with advanced treatment methods while maintaining the national movement. In terms of low-cost intervention in rural areas, yoga and Ayurvedic therapies (such as herbal hot compresses) can continue to be promoted. By taking advantage of local cultural acceptance, “standing desks” and regular break reminder systems can be implemented in IT enterprises. Non-essential spinal surgeries can be curbed, and the popularization of conservative treatments (such as physical therapy) can be enhanced (36). While ensuring drug research and development, South Africa needs to further develop in terms of the overall economy to reduce the economic burden of low back pain on patients. Screen for chronic low back pain in HIV patients (which may be caused by antiretroviral drugs or osteoporosis), provide comprehensive treatment. For areas with insufficient resources, distribute painkillers and simple rehabilitation manuals to remote areas through Mobile Clinics, strengthen the protection of the mining occupational population, and force mining companies to provide back support equipment. Have regular spinal health check-ups (37). Meanwhile, the targeted policies of the United States and Europe can also be referred to. For example, the National Institute for Occupational Safety and Health (NIOSH) in the United States promotes comprehensive health programs and encourages enterprises to prevent low back pain through exercise courses, etc. The European Union has proposed the Occupational Safety and Health Framework Directive (89/391/EEC), which requires employers to assess workplace risks (such as heavy object handling) to prevent occupational low back pain (38, 39). To sum up, population aging and high-intensity occupational burdens have become common challenges for low back pain in the BRICS countries. The older adult group has a more severe disease burden, and high-intensity occupations such as nurses and doctors also have a more severe disease burden. In this regard, we can draw on the successful policies of the United States and Europe and propose rehabilitation courses for low back pain to the BRICS group, such as stretching exercises. At the same time, employers in the BRICS countries are required to conduct intensity assessments of their workplaces. For jobs with high labor intensity, working hours can be reduced to lower the intensity.

The common problems of aging and high-intensity occupations in the burden of low back pain in the BRICS countries cannot be ignored. Globalization has made global health governance a major strategic concern worldwide, and the BRICS, which represent emerging countries, are playing an increasingly important role in it. China and Brazil, for example, are the world’s largest developing countries and BRICS members, and the direction of their cooperation in healthcare, particularly public health, will have a major impact on the global landscape (40). The importance of burden of disease research is evident, as developing countries account for 12% of the global disease burden from neglected diseases (41). Technical cooperation among the BRICS plays an important role in global health governance (42). Therefore, it is suggested that decision-makers adopt the method proposed in this paper while advancing the process of globalization to improve the common problems of low back pain in the BRICS countries.

## 5 Conclusion

The results of the research indicate that the burden of low back pain is decreasing in China and India, but morbidity and DALY values remain elevated. LBP is increasing in countries such as Russia, Brazil, and South Africa, and prevention strategies need to be strengthened. Females and the older adult are at high risk of low back pain, and occupational ergonomics, along with a high BMI, are major risk factors in BRICS countries. Projections suggest slight growth in global cases in the future, with minor improvements in male cases but a gradual increase in female cases.

## Data Availability

The original contributions presented in the study are included in the article/supplementary material, further inquiries can be directed to the corresponding author.

## Footnotes

^1^ https://ghdx.healthdata.org/gbd-2021
